# Neuro-endocrine basis for altered plasma glucose homeostasis in the Fragile X mouse

**DOI:** 10.1186/1423-0127-17-S1-S8

**Published:** 2010-08-24

**Authors:** Abdeslem El Idrissi, Xin Yan, Francoise Sidime, William L’Amoreaux

**Affiliations:** 1 Department of Biology, College of Staten Island, The City University of New York, 2800 Victory Boulevard, Staten Island, NY 10314, USA; 2 Doctoral Program in Biology – Neuroscience, The Graduate Center, The City University of New York, 365 Fifth Avenue, New York, NY 10016, USA; 3 Center for Developmental Neuroscience, College of Staten Island, The City University of New York, 2800 Victory Boulevard, Staten Island, NY 10314, USA; 4 Advanced Imaging Facility, College of Staten Island, The City University of New York, 2800 Victory Boulevard, Staten Island, NY 10314, USA

## Abstract

**Background:**

The fragile X mouse model shows an increase in seizure susceptibility, indicating an involvement of the GABAergic system via an alteration in cellular excitability. In the brain, we have previously described a reduction in GABA_A_ receptor expression as a likely basis for this susceptibility. In the brains of fragile X mice, this reduction in receptor expression culminates with a concomitant increase in the expression of glutamic acid decarboxylase (GAD), the enzyme responsible for GABA synthesis. Further, voltage-sensitive calcium channel expression is reduced in the pancreas of the fragile X mouse. Since there are considerable similarities in the GABAergic system in the brain and pancreas, we evaluated the protective role of taurine in pancreatic islet development in both wild type (WT) and fragile X mice (KO).

**Methods:**

One-month-old FVB/NJ males or age-matched fmr1-knockout (KO) mice were supplemented with taurine in drinking water (0.05% w/v) for four weeks. Age-matched controls were fed water only for the same duration. At four weeks, mice were sacrificed and pancreases processed for histology and immunohistochemical studies on changes of insulin, glucagon and somatostatin expression. Additional mice were subjected to a glucose tolerance test.

**Results:**

Taurine treatment resulted in a significant increase in the number and size of islets. WT taurine-fed mice, slightly hypoglycemic prior to glucose injection, showed significantly reduced plasma glucose at 30 min post-injection when compared to control mice. KO mice had normal baseline plasma glucose concentration; however, following glucose injection they had higher plasma glucose levels at 30 min when compared to controls. Supplementation of taurine to KO mice resulted in reduced baseline levels of plasma glucose. After glucose injection, the taurine-fed KO mice had reduced plasma glucose at 30 min compared to KO. Concomitant with the increased islets size and glucose tolerance observed in taurine-fed mice there was an increase in insulin, glucagon and somatostatin immunoreactivity in the islets of WT mice. In the KO mice however, insulin levels were not affected whereas glucagon and somatostatin levels were reduced. Exocytosis of these hormones is calcium-dependent, therefore any exacerbation of calcium homeostasis could affect hormone release. We found the expression of the voltage sensitive calcium channels (VSCC) is drastically reduced in the pancreas of fragile X mice.

**Conclusions:**

During early development, the VSCC play an important role in calcium-dependent gene expression. Since these channels are also involved in depolarization and calcium-mediated vesicular release of neurotransmitters and pancreatic hormones, alterations in the expression of VSCC not only will affect calcium-mediated gene expression but also hormonal and neurotransmitter release creating therefore a neuroendocrine perturbation in the fragile X that may potentially affect other organ systems. We find that in the fragile X mouse, taurine treatment may partially restore functionality of the neuro-endocrine pancreas.

## Background

The fragile X syndrome includes hyperarousal, hypersensitivity to sensory stimuli and an increased prevalence of seizures [1,2]. The mouse model for this disorder [3,4] is a knockout of the *fmr1* gene (fragile X mental retardation 1 gene) which has a reduction in the expression of the fragile X mental retardation protein (FMRP). This model has increased seizure susceptibility [5-7] which may be a direct parallel to elements of the syndrome that suggest reduced inhibition/increased excitability. Our investigations of the molecular basis of increased seizure susceptibility in the fragile X mouse indicate a reduction in GABA_A_ receptor expression [8]. Since these receptors play a major role in inhibition, their reduction helps explain the increased seizure susceptibility in this mouse model for fragile X and suggest that the GABAergic system may be affected in the fragile X syndrome.

We have previously described an increased expression of glutamic acid decarboxylase (GAD), the enzyme responsible for the synthesis of GABA, the neurotransmitter agonist for GABA_A_ receptors. This increase is likely a response of the brain to reduced inhibition - a response that has been observed in other models of elevated excitability [9]. The excitability of neuronal circuits is kept within a normal range through feed-forward and -back inhibition, mediated by inhibitory interneurons. These neurons continuously adjust their inhibitory output to match the level of excitatory input. Thus, when there is reduced inhibition of postsynaptic neurons, feedback from these neurons causes the presynaptic neurons to increase their inhibitory output. In the example of fragile X mouse brain, reduced GABA_A_ receptor expression on postsynaptic membranes would induce an increase in GAD expression, thus increasing the bioavailability of GABA in presynaptic terminals. Therefore, increased GAD may represent a secondary response to the direct effects of FMRP depletion.

We have previously demonstrated [10-12] that mice chronically supplemented with taurine in their drinking water showed biochemical changes in the GABAergic system similar to those observed in fragile X mouse, including a reduced GABA_A_ receptor, increased GAD expression and a lower threshold for seizure induction.

Taurine (2-aminoethanesulfonic acid) is a sulfur-containing amino acid. It is one of the most abundant free amino acids in many excitable tissues, including the brain, skeletal and cardiac muscles. Physiological actions of taurine are widespread and include bile acid conjugation, detoxification, membrane stabilization, osmoregulation, neurotransmission and modulation of cellular calcium levels [13-17]. Furthermore, taurine plays an important role in modulating glutamate and GABA neurotransmission [18-20]. We have previously shown that taurine prevents excitotoxicity in vitro primarily through modulation of intracellular calcium homeostasis [18].

Outside of the central nervous system, taurine also is essential during developmental processes. Taurine is added to milk formula and in a solution for parenteral nutrition of premature babies to prevent retinal degeneration and cholestasis [21,22]. Taurine is found at high concentrations in pancreatic islets [21] and is able to prevent pancreatic alterations induced by gestational malnutrition especially low-protein diet [23-26]. In addition, taurine administration during gestation delays the mean onset time of diabetes in non-obese diabetic (NOD) mice [27]. Taurine supplementation to dams fed a normal diet produces weak glucose intolerance and decreases islet sensitivity to cytokines in offspring [26]. Moreover, taurine participates in glucose metabolism in adults [28-30].

Previous reports show that the islets from taurine-treated mice had almost double the number of cells positive for proliferating cell nuclear antigen (PCNA). This increased proliferation was accompanied by a reduction in the incidence of apoptosis in islet cells, and also a significant increase in the number of islet cells immunopositive for IGF-II [27]. Peak of islet cell apoptosis is maximal in the rat pancreas 14 days after birth and is temporally associated with a fall in the islet cell expression of IGF-II [31]. IGF-II functions as an islet survival factor in vitro. The induction of islet cell apoptosis in vivo may involve an increased expression of inducible nitric oxide synthase (iNOS) within β-cells. Interestingly, taurine is a potent inhibitor of iNOS [32].

## Methods

*Animals:* All mice used in this study were two-month-old FVB/NJ males. For taurine-fed mice, taurine was dissolved in water at 0.05%, and this solution was made available to the mice in place of drinking water for 4 weeks beginning at 4 weeks of age. All mice were housed in groups of three in a pathogen-free room maintained on a 12-hr light/dark cycle and given food and water ad libitum. All procedures were approved by the Institutional Animal Care and Use Committee of the College of Staten Island/CUNY, and were in conformity with National Institutes of Health Guidelines. The number of mice sufficient to provide statistically reliable results was used in these studies.

*Quantification of size and number of pancreatic islets:* Two-month-old mice were perfused with 4% paraformaldehyde and pancreases were isolated attached to the pyloric region of the stomach and the duodenum. The initial part of the duodenum served to orient the pancreas for the sectional plane. Tissue was cryoprotected with 30% sucrose and cryosectioned at a thickness of 30 µm. Sections were stained with hematoxylin and eosin. Contiguous sections were stained with propidium iodide to visualize condensed chromatin as an indication of apoptotic cell death. Microscopy was performed independently by three laboratory assistants unaware of the treatment conditions.

*Immunohistochemistry:* Cryosections were made as described above and placed onto gelatin-subbed slides. Non-specific binding sites were blocked using 4% bovine serum albumin (BSA), 2% normal goat serum (NGS), and 0.05% Triton X-100 in 0.01M phosphate-buffered saline (pH 7.4). Following the blocking step, the slides were rinsed in an antibody dilution cocktail (ABD) consisting of 2% BSA and 1% NGS in 0.01M PBS. Primary antibodies (Chemicon International, Temecula CA) employed were directed against insulin (mouse host), glucagon (rabbit host), or somatostatin (rabbit host) and diluted 1:500 in ABD. For these studies, the mouse anti-insulin was paired with either rabbit anti-glucagon or rabbit anti-somatostatin. The primary antibodies were incubated overnight at 4^o^C and then unbound antibodies rinsed with ABD. Secondary antibodies were all raised in goat and directed against appropriate primary antibody type. The anti-mouse IgG was conjugated to Alexa Fluor 488 (Invitrogen/Molecular probes, Carlsbad CA) and the anti-rabbit IgG was conjugated to Cy5 (Jackson Immunological, West Grove PA). Images were obtained by confocal microscopy (Leica SP2 AOBS). To determine relative changes in protein expression, the gain and offset was identical for all comparisons. Once the images were obtained, relative fluorescence levels were identified for eight islets per treatment, with 30 regions of interest of identical dimensions set for each islet. Changes in expression were also confirmed statistically using the Imaris x 64 software (Bitplane).

*Intraperitoneal Glucose Tolerance Test:* Mice from both groups were fasted overnight (12h) and then injected intraperitoneally with 0.02 ml/g of body weight D-glucose (7.5 % stock solution in saline). Blood samples were taken by tail venesection at 0 min (just before glucose injection) and at 30min intervals after the glucose load. Glucose was measured with Ascensia Breeze portable glucose meter (Bayer, Germany). Mice were given only water during the test.

## Results

### Taurine supplementation increases the size and number of the islets of Langerhans

**Figure 1:**
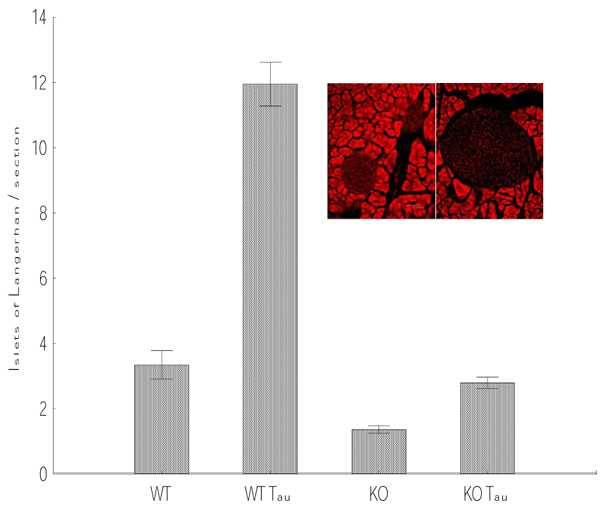
**Taurine induces an increase in the number of islets in the pancreas.** All mice were 2 months old. Taurine (0.05%) was supplemented in the drinking water. WT, n=3; KO, n=4; WT-Tau, n=3; KO-Tau, n=3. Pancreata were cryosectioned (30 µm) and stained with hematoxylin and Eosin. Each pancreas yielded approximately 150 sections. All pancreata were cut in the longitudinal plane. WT-tau had the largest number of islets per section. KO had significantly less islets than controls and treatment with taurine caused a significant increase in the number of islets. Insert shows an islet from control (left) and taurine-fed WT mouse stained with propidium iodide

Histological examination of the pancreas revealed that the KO mice had a significant reduction in the number of islets of Langerhans when compared to controls (Fig. 1; p<0.05). Furthermore, there were no noticeable histological abnormalities in the endocrine or exocrine parts of the pancreas. Surprisingly, supplementation of taurine in the drinking water resulted in a drastic and significant increase in the number of islets per section in both WT and KO mice (Fig. 1; p<0.001 for WT, p<0.01 for KO). Previously, it has been reported [27] that the islets from taurine treated mice had almost double the number of cells immunopositive for proliferating cell nuclear antigen (PCNA). This increase proliferation was accompanied by a reduction in the incidence of apoptosis in islet cells, and also a significant increase in the number of islet cells immunopositive for IGF-II. These data demonstrate that the endocrine pancreas undergoes significant modification during neonatal life, and that proliferation and apoptosis are integral mechanisms in this remodeling.

### Taurine-fed mice show hyperinsulinemia and glucose tolerance

**Figure 2:**
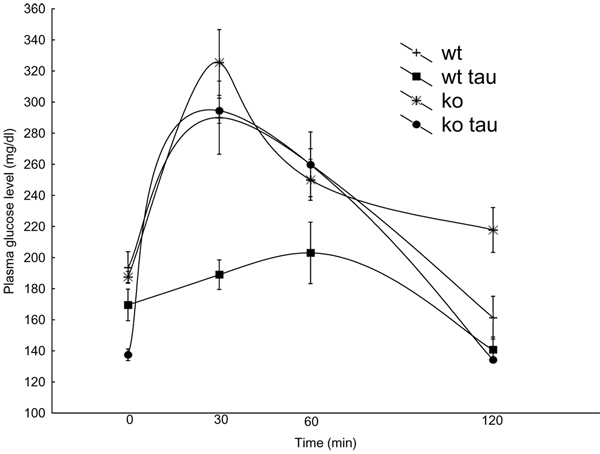
**Effect of taurine supplementation on glucose homeostasis** Intraperitoneal glucose tolerance test on overnight fasted mice. All mice were 2 months old. Taurine (0.05%) was supplemented in the drinking water. WT, n=9; KO, n=12; WT-Tau, n=10; KO-Tau, n=12.Values are expressed as means ± S.E.M obtained from three experiments.

To determine the functional significance of increased islets size and number in the pancreas of taurine-fed mice, we tested the tolerance of mice to glucose injection as an indicator of the pancreas efficiency to regulate plasma glucose homeostasis. As expected, control mice showed a drastic increase in plasma glucose concentration 30 min after challenge with a gradual decrease through 120 min. By the end of the experiment, mice were slightly hypoglycemic relative to baseline (Fig. 2).

In contrast, taurine-fed control mice showed a significant tolerance to glucose injection. Baseline plasma glucose levels indicated that these mice were slightly, but not significantly, hypoglycemic compared to controls. However, the response to glucose injection was drastically reduced (p < 0.001) at 30 min compared to controls. At 60 min following the challenge, the plasma glucose level in the taurine-fed mice rose slightly but was still significantly reduced than controls. Not until at 120 min post-challenge that the plasma glucose levels were similar in both groups. KO mice had similar baseline plasma glucose levels as WT. However, 30 min post glucose injection, glucose plasma levels were higher than those of WT. At 60 min KO plasma glucose levels were the same as WT, but two hours post injection WT plasma glucose levels continue to decline whereas the KO plasma glucose levels remained significantly higher than WT. Interestingly, when KO mice were fed taurine for four weeks prior to the glucose test they almost reacted as WT controls. Taurine supplementation induced hypoglycemia in KO mice at baseline, glucose levels were lower 30 min post injection and drastically reduced after 2 h when compared to non-taurine-fed KO mice (Fig.2).

### Taurine supplementation enhances the endocrine function of the pancreas

**Figure 3:**
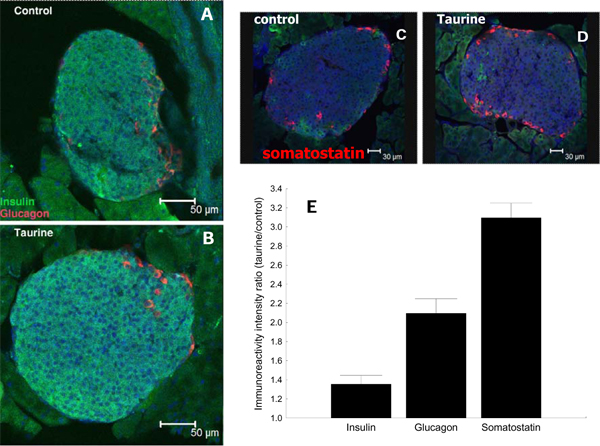
**Decreased hormonal expression in the fragile X pancreas** Effect of taurine supplementation on insulin, glucagon and somatostatin expression in pancreatic islets. A and B Representative confocal images showing insulin (Green) and glucagon (Red) immunoreactivity in pancreatic islets from control and taurine-fed mouse, respectively. C and D somatostatin immunoreactivity in the islets from a control and a taurine-fed mouse, respectively. E. Immunoreactivity intensity ratio of taurine-fed over controls.

Since plasma glucose levels are inversely correlated to the plasma insulin levels, which in turn are determined by insulin levels present in and released from the islets. Here we sought to determined a possible functional parallel between the enlargement of islet size in response to taurine supplementation, the number of islets, the resistance to glucose-induced hyperglycemia and the levels of the three main pancreatic peptides: insulin, glucagon and somatostatin. We found that the levels of these markers were significantly (p<0.01) increased in response to taurine supplementation in the drinking water. In these mice, insulin levels were increased by 50%, glucagon levels were increased by 100% and somatostatin levels by 300% (Fig. 3 A-E ). The increase in insulin level is consistent with the diminished peak of plasma glucose levels at 30 and 60 min.

### Altered pancreatic hormones in fragile X mice

**Figure 4:**
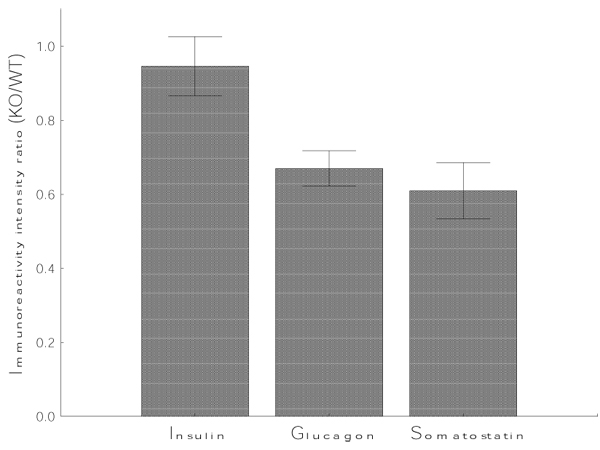
**Decreased hormonal expression in the fragile X pancreas** To determine relative changes in hormonal expression, the gain and offset was identical for WT and KO comparisons. Once the images were obtained, relative fluorescence levels were identified for eight islets per genotype, with 30 regions of interest of identical dimensions set for each islet.

In the intraperitoneal glucose tolerance test, Fragile X mice showed elevated glucose levels at 30 min when compared to WT controls (Fig. 2), suggestive of potentially altered pancreatic hormonal levels. Therefore, we measured the levels of the main pancreatic hormones by quantifying the intensity of immunoreactivity of these hormones. Interestingly we found that the levels of insulin immunoreactivity were not affected in fragile X mice pancreas whereas glucagon and somatostatin were reduced (Fig. 4).

Plasma glucose level is mainly controlled by insulin and glucagon. These two hormones are antagonistic to each other and their secretion is partially controlled by somatostatin. Reduced glucagon and somatostatin expression in the fragile X pancreas could be responsible for the altered response to the glucose tolerance test observed in the fragile X mice.

### Reduced expression of the VSCC in the pancreas of fragile X mice

**Figure 5:**
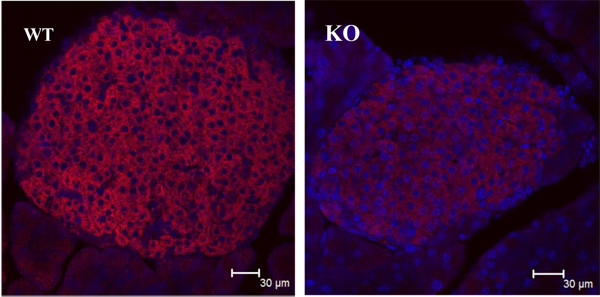
**Decreased expression of the VSCC in the fragile X pancreas** Representative confocal images showing VSCC immuno-reactivity in pancreatic islets from WT and Fmrp1-KO mice, respectively. Sections were counterstained with the nuclear stain DAPI.

In neurons, the main route for calcium entry is the activation of NMDA and VSCC. In the pancreas however, calcium enters only through VSCC expressed on the islet cells. These channels are activated through depolarization mediated by the electrogenic transport of glucose that is coupled with sodium. Thus, activation of the VSCC on the β-cells causes the co-release of GABA and insulin. GABA inhibits glucagon release while insulin lowers plasma glucose concentration. We found that fragile X mice have reduced expression of the VSCC in the islets of Langerhans (Fig. 5). Such a reduction in the expression of these channels would result in reduced calcium influx and reduced intracellular calcium necessary for vesicular release of pancreatic hormones, thus leading to the altered handling of glucose loads.

## Discussion

Histological examination of the pancreas revealed that taurine-fed mice had a significant increase in the size of islets of Langerhans when compared to controls (Fig. 1). There were no noticeable histological abnormalities in the endocrine or exocrine parts of the pancreas. Surprisingly, supplementation of taurine in the drinking water resulted in a drastic and significant increase in the number of islets per section (Fig.1). KO mice had fewer islets than WT mice and these islets were relatively smaller. Addition of taurine to the drinking water of KO mice resulted in a drastic increase in the number of islets. However, supplementation of taurine to KO mice did not induce the same level of changes observed in the WT mice. This could be due to the differential sensitivity to taurine between WT and KO mice or to efficiency of downstream signaling mechanisms activated by taurine. As PCNA and IGF-II levels increase and apoptosis decreases in the islets of taurine-fed mice, the assumption that taurine may prevent apoptotic remodeling in the developing pancreas has been proposed [27,31]. We provide evidence here that the consequence of apoptosis inhibition by taurine was an increase in the size and perhaps the number of islets. Scaglia et al., [33] have shown increased replication and decreased incidence of apoptosis in β-cells in the presence of IGF-II. Consistent with these observations, we found that supplementation of taurine in the drinking water for 4 weeks resulted in a significant increase in the number and size of islets (Fig. 1). Functionally, this may also be coupled with increases in insulin and glucagon expression as well as somatostatin levels in the islets.

Concomitant with these histological observations, we found that the immunoreactivity of the main pancreatic markers was significantly elevated following taurine supplementation (Fig. 3). Such an increase in pancreatic hormonal levels offered tight regulation of plasma glucose levels when these mice were challenged with elevated glucose levels during a glucose tolerance test (Fig. 2). The increased levels of expression of these markers that we report here were from comparison of regions of interest in the islets, and not from total number of cells in the pancreas producing the markers. We therefore hypothesize that the taurine not only lead to more islets in the pancreas but subsequently to increases in the expression of these peptides within the islet cells.

We have shown previously [34] that taurine supplementation to mice resulted in an increase of brain somatostatin levels. Here we showed that the effects of taurine on somatostatin expression are not limited to the brain but include the pancreas and potentially other somatostatin-expressing organs. The observed effects of taurine on the upregulation of somatostatin expression are not well understood and could be mediated at the transcriptional level.

The other important finding of this study is the reduced expression of VSCC. Such a drastic reduction would affect hormonal release and thus plasma glucose homeostasis.

## Conclusions

Fragile X mice have reduced expression of the VSCC (pancreas and brain, not shown here). This could potentially explain the altered plasma glucose homeostasis and altered neuronal excitability in the fragile X brain. We have, as well as others, previously shown that the function of the GABAergic system is altered in the Fmrp1-KO mice. Here, we show further that the alterations in the GABAergic system coupled with reduced expression of the VSCC may be responsible for the altered glucose homeostasis observed in the fragile X mice.

## Competing interests

The authors have no competing interests.

## Authors' contributions

AEI conceived of the study, designed the study, performed the statistical analysis and drafted the manuscript. XY and FS participated in the glucose tolerance test and performed the immunohistochemical analyses. WJL participated in the study design and coordination as well as edited the manuscript. All authors read and approved the final manuscript
